# A taxonomic review of *Aramides cajaneus* (Aves, Gruiformes, Rallidae) with notes on morphological variation in other species of the genus

**DOI:** 10.3897/zookeys.500.7685

**Published:** 2015-04-27

**Authors:** Rafael Sobral Marcondes, Luís Fábio Silveira

**Affiliations:** 1 Museu de Zoologia da Universidade de São Paulo, Seção de Aves. Caixa Postal 42494, São Paulo, SP, 04218-970, Brazil; 2 Universidade de São Paulo, Instituto de Biociências, Departamento de Zoologia, Pós-Graduação. Caixa Postal 11461, São Paulo, SP, 05422-970, Brazil; 3 Current address: Department of Biological Sciences and Museum of Natural Sciences. 119 Foster Hall, Louisiana State University, Baton Rouge, LA 70803, USA

**Keywords:** *Aramides wolfi*, *Aramides mangle*, *Aramides ypecaha*, Central America, voice, nomenclature, Wood-rails

## Abstract

The taxonomy of the polytypic and wide-ranging Gray-necked Wood-rail, *Aramides cajaneus* is reviewed, based on external morphology and voice. Throughout its distribution, there is extensive plumage variation, much of it taxonomically uninformative. However, through three informative plumage characters, as well as morphometric and vocal variation, three phylogenetic species were identified within what is today known as *Aramides cajaneus*, all of which already had available names: *Aramides albiventris* Lawrence, 1868, from southern Mexico to northeastern Costa Rica, *Aramides cajaneus* (Statius Müller, 1776) (*sensu stricto*), from southwestern Costa Rica to Argentina, and *Aramides avicenniae* Stotz, 1992, from a small section of the coast of southeastern Brazil. *Aramides albiventris* presents extensive plumage variation, but with no geographic structure. The song of *Aramides cajaneus* and *Aramides avicenniae* is strikingly and completely different from the song of *Aramides albiventris*. A previously unnoticed parapatric pattern of distribution of *Aramides cajaneus* and its congener *Aramides saracura* in southeastern Brazil is described, and we clarify that the name *Aramides plumbeicollis*, included in the synonymy of *Aramides albiventris*, was first made available in 1892, rather than in 1888 as is widely referred. In addition, plumage variation in *Aramides ypecaha*, *Aramides wolfi*, and *Aramides mangle* is discussed.

## Introduction

The genus *Aramides* (Rallidae), as currently accepted, includes seven species of medium to large rails inhabiting mainly aquatic and semi-aquatic environments throughout most of the Neotropics. They have long bills and legs, mostly gray, black, brown and green plumage, barred underwing coverts and a black tail. Of all the species in the genus the Gray-necked Wood-rail, *Aramides cajaneus* (Statius Müller, 1776), is the most widespread and is found from Mexico to Argentina. It is diagnosable by having an entirely gray neck, which contrasts with its chestnut chest (Ripley 1977, Taylor 1996, Sick 1997, Taylor 1998). However, its plumage is highly variable, especially regarding the colors of the nape, lower chest and back, which led to it currently being recognized as containing nine subspecies, making it the only polytypic species in the genus (Bangs 1907, Hellmayr 1929, Hellmayr and Conover 1942, Ripley 1977, Stotz 1992, Taylor 1996, Taylor 1998).

The taxonomic history of *Aramides cajaneus* is rife with disagreements concerning the allocation of specific or subspecific status to populations, as well as about the morphological characters, diagnoses and geographic limits of these putative taxa. Statius Müller (1776) described *Fulica Cajanea*, based on the bird named “Poule d’Eau de Cayenne” (Cayenne’s water hen), illustrated on plate 352 of Daubenton’s (1765–1781) Planches Enlumineés d’Histoire Naturelle. This taxon was included by Pucheran (1845) in his newly described genus *Aramides*, and thereafter became known as *Aramides cajanea*. David and Gosselin (2011) drew attention to the fact that *Aramides* is masculine, whilst “*Cajanea*”, as intended by Statius Müller, is an adjective. Thus the correct agreement is *cajaneus*.

The nine subspecies of *Aramides cajaneus* can be divided into two groups. The first consists of five subspecies usually considered more closely related to *Aramides cajaneus albiventris*, and that occur from Costa Rica northwards. It includes *Aramides cajaneus albiventris*, *plumbeicollis*, *mexicanus*, *pacificus* and *vanrossemi*. The first to be described was *Aramides albiventris*, from Belize and Guatemala, by Lawrence (1868). *Aramides plumbeicollis* was then described by Zéledon (1892) from northeastern Costa Rica. At the time of their descriptions, both were considered allied to, but separate species from *Aramides cajaneus*. *Aramides albiventris* was distinguished from *cajaneus* by its paler chest, black belly and presence of a white band in the lower chest. *plumbeicollis* was distinguished from both *cajaneus* and *albiventris* by its russet mantle. Later, Bangs (1907) considered *plumbeicollis* a subspecies of *albiventris*, and described a new subspecies, *Aramides albiventris mexicanus*, from Vera Cruz, Mexico. This would be separable from nominal *albiventris* by its overall darker coloration and less distinct white band in the lower chest, but the two subspecies reportedly showed a certain degree of intergradation in Yucatán and Honduras. Miller and Griscom (1921) questioned this intergradation, elevated both *mexicanus* and *plumbeicollis* to full species, and described *Aramides plumbeicollis pacificus* from Tipitapa, in western Nicaragua, based on its darker overall color and lack of white in the lower chest. The last of the group to be described was *Aramides vanrossemi* Dickey, 1929, from El Salvador. It would be distinguished from *albiventris* by its overall paler coloration and green rather than yellow terminal third of the maxilla. Then, for the first time and without presenting any rationale, Peters (1934) and later Hellmayr and Conover (1942) considered all the above-mentioned taxa to be subspecies of *Aramides cajaneus*, a treatment that has been followed by all authors ever since.

The second group of subspecies consists of *Aramides cajaneus cajaneus* and the three taxa considered more closely related to it, namely *Aramides cajanea latens*, *morrissoni* and *avicenniae*. They are distributed from Costa Rica southwards. *Aramides cajaneus cajaneus* occurs in southern Costa Rica, Panama, and throughout most of South America east of the Andes, except where it is replaced by *Aramides cajanea avicenniae* (see below). *Aramides cajanea latens* was described by Bangs and Penard in 1918 and *Aramides cajaneus morrissoni* by Wetmore in 1945. Both are from the Pearl Islands archipelago, off the Pacific coast of Panama, with *latens* found on the islands of San Miguel and Viveros, and *morrissoni* on San José and Pedro González. They would be distinguished from *cajaneus* and from each other by subtle differences in size and overall coloration. The final subspecies, *Aramides cajanea avicenniae* was described by Stotz in 1992, from the coast of São Paulo state, southeastern Brazil, based on it having a gray, instead of green, back.

*Aramides cajaneus cajaneus* has several junior synonyms, erected on the basis of one or very few specimens: *Aramides cajanea venezuelensis* Cory, 1915, *Aramides cajanea peruviana* Cory, 1915, *Aramides cajanea salmoni* Chubb, 1918 and *Aramides cajanea grahami* Chubb, 1919. None of these, however, was ever accepted as valid after their publication. Another form which has been considered a junior synonym is *Aramides chiricote*, from Paraguay, first described as *Rallus chiricote* by Vieillot (1819) based on Azara’s (1805) “*chiricóte*”. Unlike the aforementioned names, it did receive consideration in the literature, being recognized as a subspecies by Sharpe (1894), and having its validity discussed, but discarded, by Bangs (1907), Hellmayr (1906, 1929), Hellmayr and Conover (1942) and Stotz (1992). Yet another taxon related to *Aramides cajaneus* is *Aramides gutturalis* Sharpe, 1894, based on a single peculiar specimen of uncertain provenance. It was accepted as a full species by Peters (1934) and Hellmayr and Conover (1942), but has since been considered a badly prepared skin of *Aramides cajaneus* (Meyer de Schauensee 1966, Taylor 1996, 1998).

In contrast to *Aramides cajaneus*, all other species of *Aramides* are monotypic and have much more restricted distributions. They are also among the least known species of Neotropical rails. Basic descriptive data, such as voice and distribution, are deficient or lacking for some of them (Ripley 1977, Taylor 1998, Vaca et al. 2006, Redies 2010, Karubian et al. 2011). Most significantly, none of them has ever had its morphological variation analyzed.

In light of its complex taxonomic history and the extensive variation in external morphology presented by *Aramides cajaneus*, its plumage and morphometric variation is reviewed and its vocalizations examined in a taxonomic context for the first time. Based on these data, a revised, more adequate taxonomic treatment is proposed for the taxa currently included in it. Plumage variation in some other species of *Aramides* is briefly presented and discussed for the first time.

## Material and methods

800 skins of *Aramides cajaneus* were examine by the authors, including representatives of all its subspecies, deposited in the following institutions: Museu de Zoologia da Universidade de São Paulo (MZUSP), São Paulo, Brazil; Museu Nacional da Universidade Federal do Rio de Janeiro (MNRJ), Rio de Janeiro, Brazil; Museu Paraense Emilio Goeldi (MPEG), Belém, Brazil; Museu de História Natural do Capão da Imbuia (MHNCI), Curitiba, Brazil; American Museum of Natural History (AMNH), New York, USA; Field Museum of Natural History (FMNH), Chicago, USA; Natural History Museum (BMNH), Tring, UK; Muséum National d’Histoire Naturelle (MNHN), Paris, France; and Museum für Naturkunde (ZMB), Berlin, Germany. We examined only through photographs a further 206 specimens, deposited in the following institutions: Instituto Nacional de Pesquisas da Amazônia (INPA), Manaus, Brazil; Museu de Biologia Prof. Mello Leitão (MBML), Santa Teresa, Brazil; Museo de La Salle (MLS), Bogotá, Colombia; Colección Ornitológica Phelps (COP), Caracas, Venezuela; Carnegie Museum of Natural History (CMNH), Pittsburgh, USA; Museum of Comparative Zoology (MCZ), Cambridge, USA; National Museum of Natural History (USNM), Washington, USA; and University of California Donald R. Dickey Bird and Mammal Collection (UCLA), Los Angeles, USA. Photographs were not taken under standardized lighting conditions, but extensive experience with physical examination of *Aramides* skins (as well as of a wealth of other bird taxa) in many lighting conditions allowed us to confidently discern those photographs that allowed meaningful comparison of plumage from those that did not, and the latter were discarded from the analyses.

A list of all specimens examined, with their locality data, is available online as “Suppl. material 1: Specimens examined”. Among the specimens examined, either in person or through photographs, are the name-bearing type specimens of all the nominal taxa related to *Aramides cajaneus* mentioned above, except *Aramides chiricote* (Vieillot 1819). The holotype of *Aramides cajaneus* (Statius Müller 1776) is the bird illustrated in Daubenton’s (1765–1781) plate “Poule d’Eau de Cayenne”, and it is not known if it has been preserved as a specimen.

In addition to specimens of *Aramides cajaneus*, we also examined in person or though photographs 410 skins belonging to all other species of the genus. These were deposited in the same institutions listed above, except for a skin of *Aramides calopterus* in the Naturhistoriska Riksmuseet (NRM), Stockholm, Sweden and a skin of *Aramides wolfi* (holotype) in the Muzeum i Instytut Zoologii (MIZ), Warsaw, Poland.

Skins of all species of *Aramides* were qualitatively compared, searching for variation in pattern and color of all plumage regions. To describe colors, color names (capitalized in the text below) and codes from Munsell (1994) were sometimes used. Wing, tail, tarsus and bill height, length and width for *Aramides cajaneus* skins were all measured, following Baldwin et al. (1931). After delimitating diagnosable units in the *Aramides cajaneus* complex (see below), morphometric differences between them were assessed through analysis of variance (ANOVA) or its non-parametric counterpart, Kruskall-Wallis’ test. These were followed by the post-hoc multiple comparisons tests Tukey and Dunn’s, respectively. The level of significance (α) adopted for all tests was 0.05. To evaluate geographical variation in measurements, they were plotted against latitude and longitude. All statistical analyses were performed using GraphPad Prism 5 (GraphPad Software 2007) or SPSS 13.0 (SPSS, Inc. 2004). All qualitative and quantitative examinations of skin specimens were conducted by the first author.

92 recordings of *Aramides cajaneus* vocalizations were also analyzed from within the distribution of five of the nine subspecies. These were mostly songs, recognized by being emitted in duets and being louder and more prolonged than other vocalizations in the species’ repertoire. They were obtained from sound archives, namely Macaulay Library, Cornell University, Ithaca, USA (LNS); Fonoteca Neotropical Jacques Vieillard, Universidade Estadual de Campinas, Campinas, Brazil (FNJV); Arquivo Sonoro da Seção de Aves do Museu de Zoologia da Universidade de São Paulo, São Paulo, Brazil (MZUSP); and Wiki Aves (WA, www.wikiaves.com.br); from published compilations (López-Lánus 2009 and Minns et al. 2009), through contribution from collaborators (see acknowledgements); and from the authors’ own personal archives. Recordings were analyzed through aural inspection and, for those of good quality, as spectrograms on Raven Pro 1.4 (Bioacoustics Research Program 2011). In each recording, we measured six to nine notes for peak frequency (PF), maximum frequency (MaF), minimum frequency (MiF), bandwidth (BW), and duration (D). These measurements were taken using a frequency resolution of 46.9 Hz and time resolution of 1.06 miliseconds and are presented as mean ± standard deviation. All qualitative and quantitative (measurements) analyses of sound recordings were conducted by the first author. A list of all recordings examined is available online as “Suppl. material 2: Recordings examined”.

We adopt the General Lineage Species Concept (GLSC; de Queiroz 1998, 2005), which defines species as “lineages of metapopulations evolving separately”. This concept acknowledges that speciation is a prolonged process during which the diverging lineages acquire properties (such as diagnosability, reciprocal monophyly, reproductive incompatibility) that can be used in practice for their recognition as distinct species (de Queiroz 1998, 2005). Here, we investigate if such properties can be identified in any subpopulations of what is today understood as *Aramides cajaneus*. We focus mainly on phenotypic differentiation and diagnosability, and also consider reproductive incompatibility, inasmuch as it can be inferred from differences in song, which plays a major role in avian mating (Catchpole and Slater 1995, Baptista and Kroodsma 2001).

The lists of names in each species account include only the names applicable to each taxon and are thus strictly synonymies, not chresonymies (Dubois 2000). In other words, they do not include variants of spelling or concordance, or different combinations of genus and variations of taxonomic level (specific or subspecific) in the usage of the names. Species diagnoses are given only in relation to the other species in the *Aramides cajaneus* complex.

## Results and discussion

*Aramides cajaneus* presents extensive plumage variation throughout its vast range. However, much of this variation is not geographically structured, such that specimens from the same locality are frequently more variable between each other than they are in relation to specimens from a distant locality. These characters are, therefore, not taxonomically informative. An example of this is the chest color, which ranges from Dark Yellowish Brown (10YR 4/6) to Strong Brown (7.5YR 4/6), and varies widely within the same localities, for instance Chapada, Brazil (AMNH 34809 and 58674) and Sarayacu, Peru (AMNH 237512 to 237520). Another example is the amount of greenish or brownish coloration on the rump. For example, in specimens from Lago do Baptista, Brazil, this ranges from totally black (e. g. MZUSP 20923 and 21975) to almost totally brownish green (e. g. MZUSP 21825 and 21803), with several intermediates (e. g. MZUSP 21914 and 22008).

**Figure 1.**
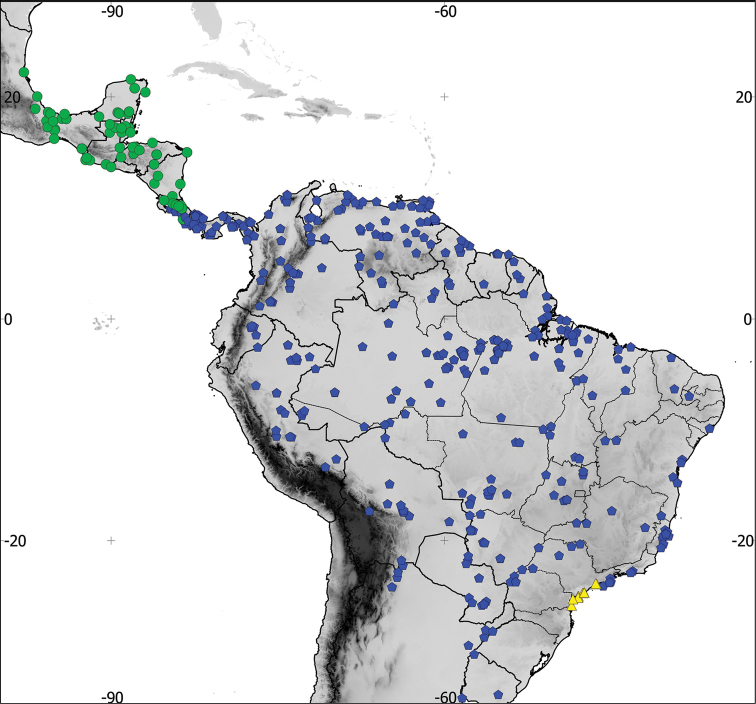
Distribution of the recognized species in the *Aramides cajaneus* complex, based on examined skins. Green: *Aramides albiventris* (Central American component), blue: *Aramides cajaneus* and yellow: *Aramides avicenniae* (South American component).

**Figure 2.**
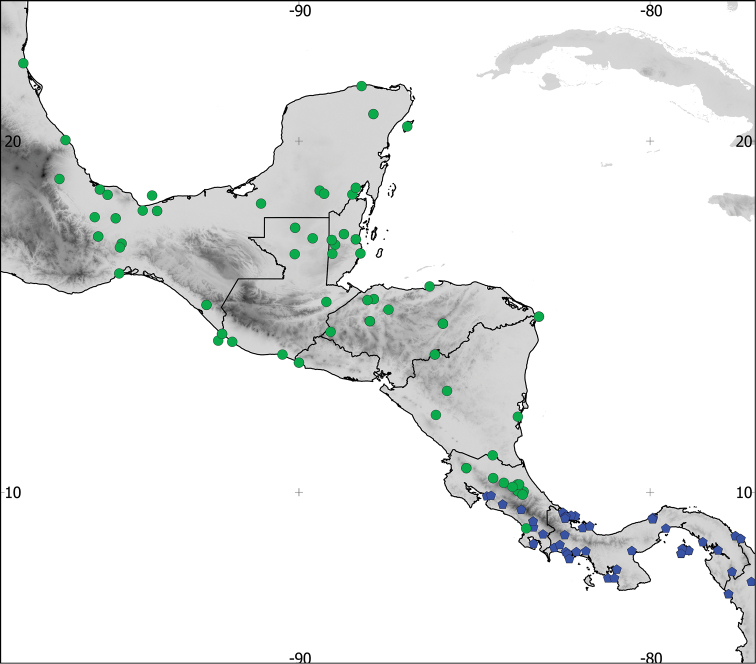
Detail of the distribution of the recognized species in Central America, based on examined skins. Green: *Aramides albiventris*, blue: *Aramides cajaneus*.

Nevertheless, three plumage characters do vary geographically and allow the delineation of diagnosable clusters of individuals. These are: (1) back color, including the presence and intensity of a brown upper back (mantle); (2) presence of white feathers in the lower chest, separating the chestnut upper chest from the black belly; and (3) presence and intensity of a brown spot in the occiput. Some of the recognized species can also be diagnosed based on remarkable geographical variation in song. Morphometric variation further contributes to characterize them, even though not to their diagnoses, because there is considerable overlap in measurements. Based on these geographically-varying plumage and voice characters, we recognize three species in the *Aramides cajaneus* complex: *Aramides albiventris*, *Aramides cajaneus*, and *Aramides avicenniae* (Figures 1 and 2). In the next sections, we detail the geographical variation in plumage, as well as in vocalizations and morphometry, and discuss the more adequate taxonomy treatments, first by establishing the very well-marked division of the complex into Central American and South American components and then, by delving into variation within each of these components.

### Division of the *Aramides cajaneus* species complex into Central American and South American components

**Figure 3.**
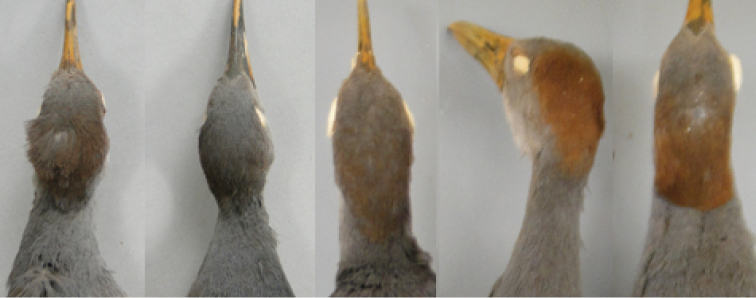
Nape of South American (the three leftmost specimens) and Central American (the two other specimens) representatives of the *Aramides cajaneus* species complex. Note the much stronger color in the latter.

**Figure 4.**
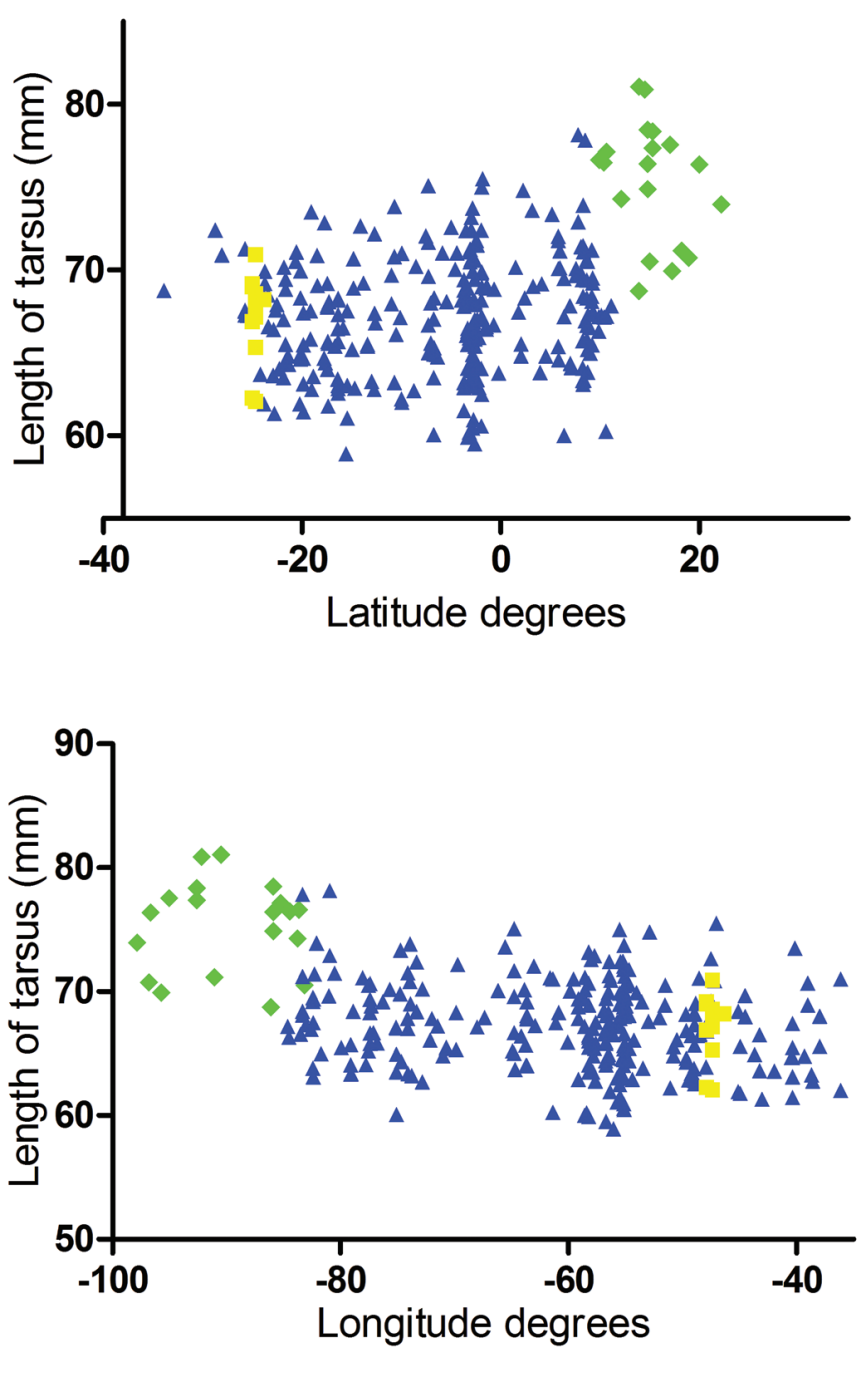
Length of tarsus of specimens in the *Aramides cajaneus* complex plotted against latitude and longitude. Green: *Aramides albiventris*; blue: *Aramides cajaneus*; yellow: *Aramides avicenniae*. Note the discontinuity in variation around latitude 10° N and longitude 83° W, in Costa Rica, where the distributions of *Aramides cajaneus* and *Aramides albiventris* abut each other.

Plumage, vocal and morphometric characters support a clear split between a Central American component (from Mexico south to Costa Rica) and a mainly South American (also including Panama, part of Costa Rica and the Pearl Islands) component in this species complex. In plumage, these components are distinguished from each other, without intermediates, by the much stronger-colored brown nape of Central American birds (Figure 3). Morphometrically, there is an evident discontinuity in variation of bill and tarsus length around 10°N and 83°W, in Costa Rica, where the two components substitute each other (Figure 4). (Other measurements, when plotted against latitude and longitude, did not show any discernable pattern in variation, and these plots are therefore not shown.) Descriptive statistics for each recognized taxon are presented on Table 1.

**Table 1.** Mean ± standard deviation (first line), range (second line), and sample size (third line) of morphometric variables for each sex of each of the recognized species.

Taxon	Sex	Wing	Tail	Tarsus	Bill height	Bill width	Bill length
*Aramides cajaneus*	Males	184.4±7.98	65.19±5.73	67.22±3.51	11.36±0.74	5.26±0.43	52.23±3.19
		159–206	50.51–82.69	58.92–78.16	9.01–13.37	3.70–6.50	38.53–59.90
		277	260	279	231	274	280
	Females	179.0±7.98	63.02±6.10	65.07±4.26	10.86±0.71	5.05±0.46	50.19±2.83
		155–202	49.69–85.79	47.70–76.59	9.02–13.22	3.72–6.94	43.20–59.07
		223	216	224	192	224	223
*Aramides avicenniae*	Males	189.4±7.00	66.22±3.15	67.07±2.80	12.56±0.65	5.73±0.42	54.74±1.91
		180–200	59.32–70.80	62.10–70.94	11.6–13.8	5.00–6.20	53.48–56.10
		11	11	11	7	8	10
	Females	182.7±9.18	66.10±6.83	65.15±2.34	11.86±0.62	5.20±0.39	51.39±0.93
		170–195	56.23–75.30	60.20–68.20	11.00–12.62	4.59–5.64	48.70–54.69
		7	9	9	6	7	9
*Aramides albiventris*	Males	186.9±7.59	58.19±539	75.33±3.54	11.91±0.84	5.43±0.58	63.40±4.23
		173–201	51.04–68.81	68.74–81.06	10.34–12.76	4.26–6.21	54.24–71.06
		18	18	20	11	20	20
	Females	179.05±8.35	57.80±6.51	72.81±4.01	11.16±0.40	5.22±0.34	60.54±4.39
		166–196	48.97–69.07	67.42–80.25	11.54–11.95	4.59–5.88	53.60–68.22
		17	13	19	14	19	17

**Figure 5.**
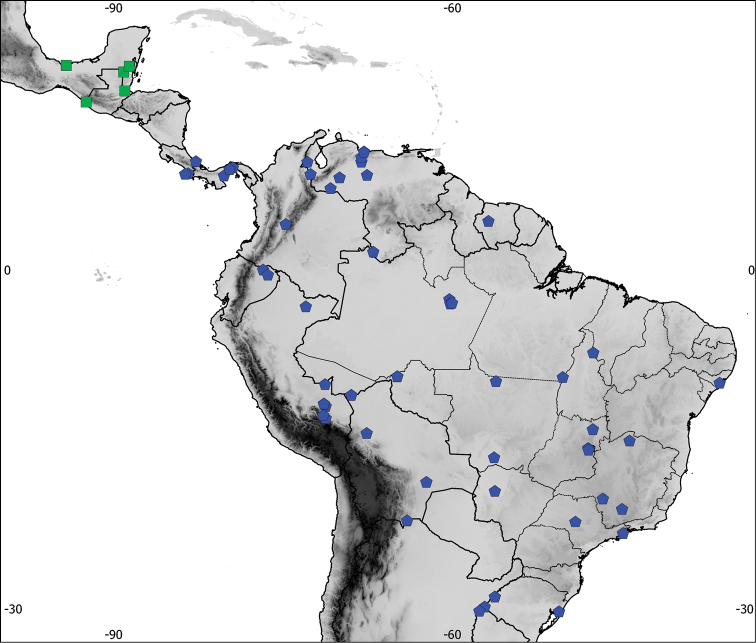
Distribution of the analyzed song recordings of the South American (blue), and Central American (green) components of the *Aramides cajaneus* species complex. Their songs are strikingly different; see text for details.

**Figure 6.**
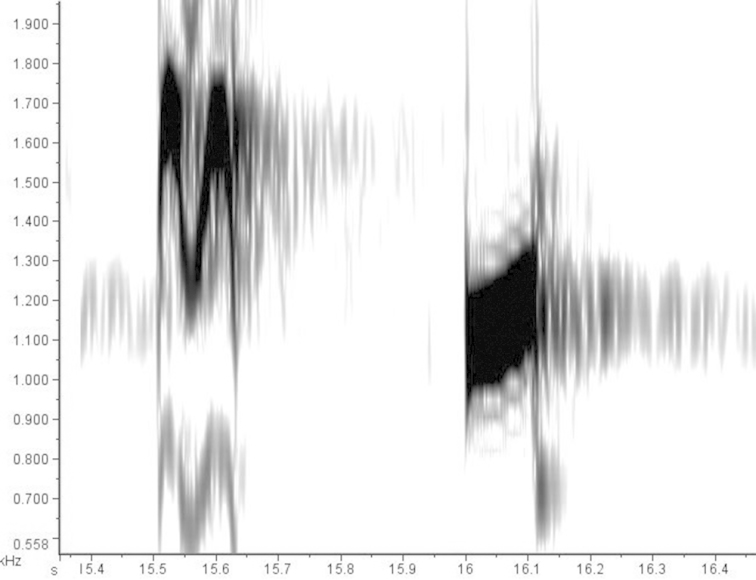
Spectrogram of the typical phrase of the song of the South American component of the *Aramides cajaneus* species complex (LNS 51765). Note that this spectrogram is not in the same scale as the spectrogram in Figure 7.

The differences in song are most striking. All available recordings from South America, Panama, and the Caribbean side of Costa Rica (Figure 5), corresponding to the South American component, show a song with a basic phrase consisting of two notes (Figures 6). The first note has ascending-descending-ascending-descending frequency modulation, giving it the approximate shape of an “M” in a spectrogram (PF: 1603±66.21 Hz; MaF: 1946±153.5 Hz; MiF: 939.2±96.39 Hz; BW: 1007±178.3 Hz; D: 0.154±0.0288 sec). The second note is shorter, has a lower frequency, and appears in spectrograms as a simple, slightly ascending line (PF: 1170±87.12 Hz; MaF: 1372±112.4 Hz; MiF: 924.6±75.7 Hz; BW: 447.8±83.24 Hz; D: 0.1146±0.0269 sec). In a typical song bout, performed in a duet, this two-note phrase is repeated in a quick, loud, and lengthy sequence, occasionally interrupted by a short series of lower-pitched notes.

**Figure 7.**
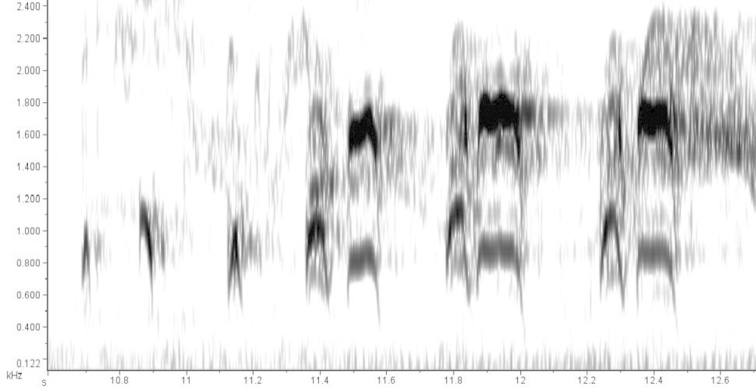
Spectrogram of the typical phrase of the song of the Central American component of the *Aramides cajaneus* species complex (LNS 23152). Note that this spectrogram is not in the same scale as the spectrogram in Figure 6.

Songs from Belize and southern Mexico (Figure 5), in the range of the second component, are strikingly different (Figure 7). The basic phrase consists of three to four short introductory notes of ascending-descending modulation, followed by three pairs of notes, with similar frequency modulation. In each pair, the first note (PF: 1246±293.92 Hz; MaF: 1436±259.7 Hz; MiF: 878±427.23 Hz; BW: 558±167.5 Hz; D: 0.067±0.014 sec) is shorter than the second (PF: 2037±368.2 Hz; MaF: 2294±358.42 Hz; MiF: 1189±322 Hz; BW: 1104±36.42 Hz; D: 0.111±0.033 sec). Although the sample size is limited, it seems that in a typical session of vocalizations, phrases are delivered in much longer intervals than in the first song type.

Even though only five recordings of the Central American component were available, the difference between its song and the song of the South American component is striking and consistent. There are neither intermediates nor any elements in each component’s vocal repertoires that are even remotely similar to the other’s song. In fact, the songs are so distinct that it is impossible even to draw correspondences or hypotheses of homology between their constituent notes. The difference is comparable to that observed between the songs of *Aramides cajaneus* and other species in the genus, such as *Aramides saracura* or *Aramides ypecaha*. Together with the plumage and morphometric differences, this substantiates the recognition of the Central American and South American components as distinct species-level taxa.

The two components are segregated by the Costa Rican mountain ranges, part of the Chorotega Volcanic Front (CVF) that divides lower Central America into Caribbean and Pacific catchments. This is congruent with the identification of the CVF as the location of a major phylogeographic break for several animal taxa in lower Central America (Bagley and Johnson 2014). In addition, the Costa Rican mountains are known to segregate several sister taxa of birds, such as *Amazilia decora* and *Amazilia amabilis* (Trochilidae), *Pteroglossus torquatus* and *Pteroglossus frantzii* (Ramphastidae), *Carpodectes nitidus* and *Carpodectes antoniae* (Cotingidae), among others (Zeledón 1892, Stiles and Skutch 1994).

There is one specimen that could potentially falsify the parapatric pattern described above. FMNH 30363 is clearly assignable to the Central American component, having a strong brown nape, but is labeled as coming from El Pozo, Puntarenas province, in the Pacific side of Costa Rica, where only birds belonging to the South American component are supposed to be found. There is reason, however, to believe that this specimen has been mislabeled. It, as well as a typically South America component specimen (FMNH 30364), was collected, according to their labels, by M. A. Carriker in 1907. The label of FMNH 30364 has the precise day and month of collection (June 29), but the label of FMNH 30363 has only the year, which already suggests that there may have been some sort of confusion and loss of information between its collection and its final labeling at the FMNH. Adding to the suspicion that this specimen was not collected in El Pozo is the fact that in 1910 Carriker published an annotated list of the birds of Costa Rica in which he recounts having indeed collected in El Pozo in June 1907. Curiously, however, under *Aramides albiventris plumbeicollis*, where this specimencould be expected to have been listed, he lists several specimens, but none coming from El Pozo. Besides, he writes about this taxon: “Confined entirely to the Caribbean lowlands, and probably only in the northeastern part, since there are no records of its presence in southeastern Costa Rica”. If Carriker had indeed collected a specimen with characters of *Aramides albiventris plumbeicollis* in the Pacific side of Costa Rica just three years earlier, it is very unlikely that he would fail to list it, and write that the taxon is found only in the Caribbean lowlands. Therefore, the information on the label of FMNH 30363, including locality data, is under suspicion, and this specimen does not falsify the role of the Chorotega Volcanic Front in segregating the *Aramides cajaneus* species complex into two components.

### Variation and taxonomy in the Central American component

**Figure 8.**
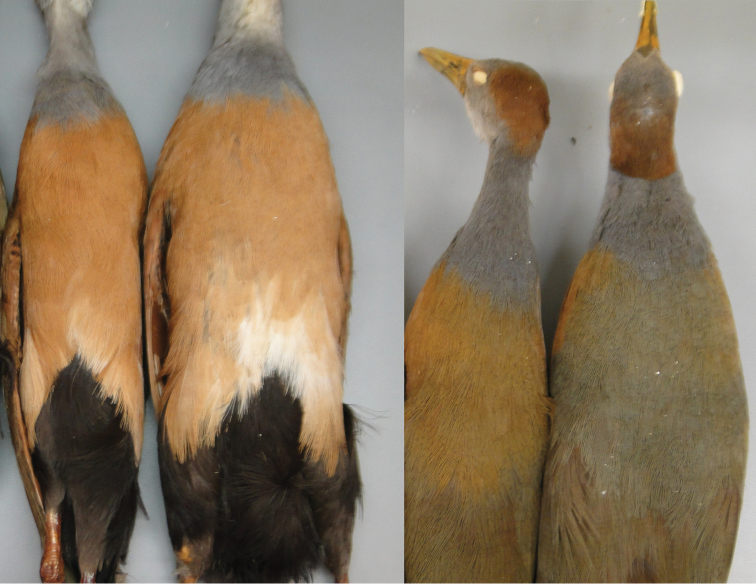
Left: Ventral view of typical specimens of the Central American morphotypes 2 (AMNH 103264) and 1 (AMNH 776255), respectively. Right: dorsal view of same specimens.

Two basic plumage morphotypes can be recognized in the Central American constituent of the *Aramides cajaneus* species complex (Figure 8). In Morphotype 1, found from western Honduras northwestwards to the extreme of the complex’s distribution in Mexico, there never is an homogeneous, conspicuous brown mantle, even though some birds do have a dull brown mantle, fainter along the midline, and there always are white feathers in the lower chest, in variable extension. In contrast, in Morphotype 2, found from eastern Honduras southeast to the Caribbean side of Costa Rica, there always is a homogeneous brown mantle, and there never are any white feathers on the lower chest, even though some birds do have in that area paler feathers than in the mid and upper chest, but not white. The characters of Morphotype 1, as well as comparison with type specimens, reveal that it is referable to *Aramides albiventris* Lawrence (syntypes from Belize and Guatemala). Morphotype 2, on the other hand, agrees with the description and holotype of *Aramides plumbeicollis* Zeledón (type locality: Jimenez, Costa Rica).

**Figure 9.**
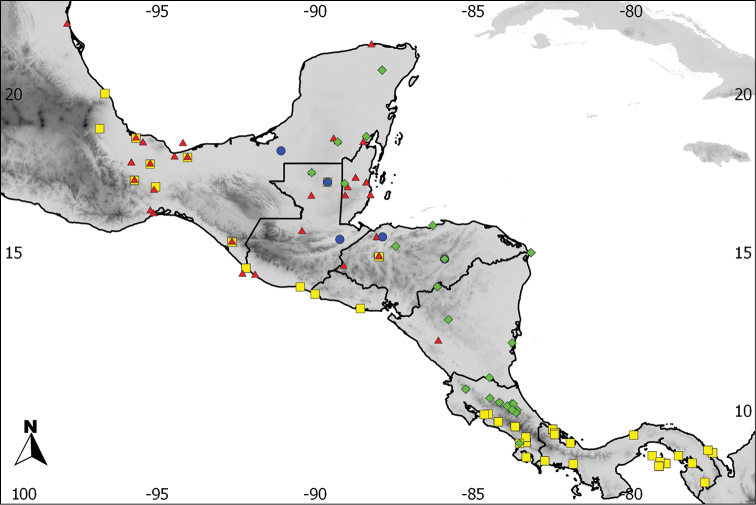
Mapping of the variation in the mantle of individuals of the *Aramides cajaneus* complex in Central America. Yellow: the upper back has no distinct coloration in relation to the mid and lower back. Red: a faint brownish coloration is present in the sides of the upper back. Blue: a faint brownish tinge is present across the upper back. Green: a complete, conspicuous brownish-orange mantle is present. Notice the lack of any discernable pattern in variation (see text for details).

**Figure 10.**
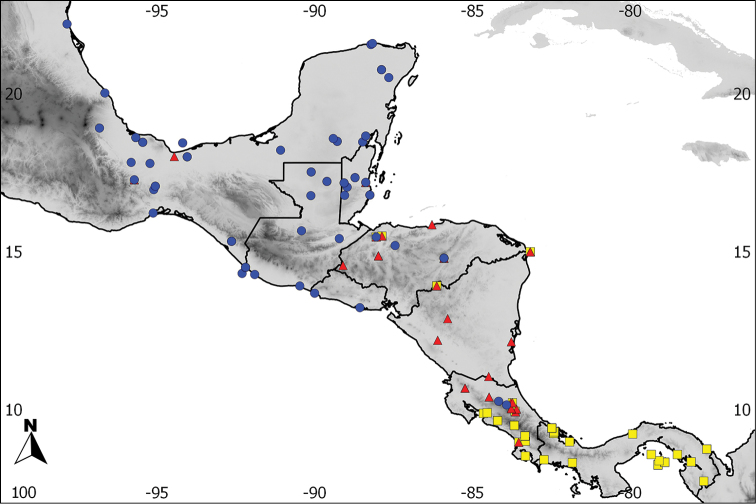
Mapping of the variation in the lower chest of individuals of the *Aramides cajaneus* complex in Central America. Yellow: no white or paler feathers in the lower chest. Red: paler chestnut, but not white, feathers are present in the lower chest. Blue: white feathers present in the lower chest.

However, in spite of the characters noted above, the distinction between the two morphotypes is doubtful and their recognition as distinct taxa is not warranted, because there are many specimens that blend characters of the two, in various combinations. Some, such as AMNH 393516, from Ocos, Guatemala, have the white chest feathers of Morphotype 1, and the full chestnut mantle of Morphotype 2. Conversely, others, such as AMNH 471954, from Mts. La Cumbre, Honduras, lack both the white lower chest feathers and the chestnut mantle. These intermediate specimens are found mainly in Honduras, Guatemala and Belize, and Quintana Roo, Campeche and Yucatán states in southwestern Mexico but also, in fewer numbers, further northwest (four specimens in Vera Cruz and Oaxaca) and south (two specimens in Costa Rica). In many cases, the intermediate specimens occur in the same localities as either “pure” morphotype, or even the two morphotypes and intermediates all together, such as in El Boquerón, in center-eastern Honduras. No particular geographic pattern of plumage variation is noticeable throughout the extensive area of intergradation (Figures 9 and 10).

Occurrence of intermediates or hybrids, by itself, does not preclude recognition of two populations as separate species, as long as the variation is not clinal and specimens from outside the intergradation zone maintain their diagnosability (Helbig et al. 2002). In the present case, plumage variation does not appear to be clinal (even though tarsus and culmen measurements, when plotted against latitude and longitude, do hint at clinal variation, the length of both increasing towards north and west; Figure 4). However, the zone of intergradation is too extensive, and intergrades too numerous, to allow recognition of two evolutionarily units. Also due to these intermediates, diagnosability between Morphotypes 1 and 2 is not absolute anywhere in Central America. Unfortunately, no songs from within the range of Morphotype 2 were available, so vocal data cannot be used to inform a decision on the taxonomic status of these populations. Therefore, based on the data we currently have at hand, it appears that the two morphotypes are deeply connected, and cannot be considered distinct evolutionary nor taxonomic units. Thus, we propose that the Central American component of the *Aramides cajaneus* species complex be recognized as a single species, *Aramides albiventris* Lawrence, 1868, with *Aramides plumbeicollis* Zeledón, 1892 (see ahead for a discussion on the date of its publication) as a junior synonym. At the same time, we also emphasize the importance of further study of these populations, in order to better understand the genetic and historical processes underlying this very complex scenario of phenotypic variation.

Regarding the other subspecies currently recognized in Central America, Miller and Griscom (1921) described *Aramides plumbeicollis pacificus*, based on a single specimen (AMNH 143684) from western Nicaragua. This specimen presents a slight indication of a chestnut mantle, but it is notably more tenuous along the midline, and it has no pure white feathers on the lower chest. It is one of the intermediate specimens between the two *Aramides albiventris* morphotypes, and thus *Aramides plumbeicollis pacificus* is a synonym of *Aramides albiventris*.

The characters used by Bangs (1907) and (Dickey 1929) to describe, respectively, *Aramides albiventris mexicanus* and *Aramides vanrossemi* do not support the recognition of these taxa when a large series of specimens is examined. Their supposed diagnostic characters in relation to *albiventris* vary widely throughout southern Mexico and Guatemala. For example, one of the putative diagnostic characters of *Aramides mexicanus* would be a narrower and more fulvous (instead of white) band in the lower chest. However, the extension and exact tone of the pale feathers in the lower chest are variable throughout the distribution of *Aramides albiventris*. AMNH 393517, from Ocos (Guatemala), for example, presents a wide, pure white band, while AMNH 393518, from the same locality, presents only a few pure white feathers, the rest of the band being yellowish white, and is in this respect very similar to AMNH 471952, from northern Vera Cruz (within the supposed distribution of *Aramides mexicanus*) and FMNH 110121, from northern Guatemala. Two specimens from Sarabia, Oaxaca (AMNH 776255 and 776256), also within the supposed distribution of *Aramides mexicanus*, are very different from each other in the amount of white feathers in the lower chest. Therefore, this character is too variable in southern Mexico and adjacent regions to be taxonomically informative. A similar situation is presented by the other putative diagnostic character of *Aramides mexicanus*, “all the colors darker” (Bangs 1907). In fact, the holotype of *Aramides mexicanus* (MCZ 102281) does not in any way stand out from the range of individual variation observed in *Aramides albiventris*, and they are therefore synonyms.

Dickey (1929) described *Aramides vanrossemi* based on a single specimen (UCLA 18750) from Barra de Santiago, Ahuachapan, El Salvador. This specimen, too, does not depart significantly from the range of individual variation seem throughout the range of *Aramides albiventris*. Contrary to the stated by Dickey, it is not “slightly paler throughout”. Also, the author’s statement that it had “lake red instead of yellow” irises is unjustified, given that all birds in the *Aramides cajaneus* complex have red irises, as attested by specimen labels and abundant photographs available online (Internet Bird Collection; http://ibc.lynxeds.com/). Similarly, the statement that the “terminal third of the maxilla [is] green instead of yellow” does not make sense as this too is typical of the whole complex. Thus, *Aramides vanrossemi* is also here considered a junior synonym of *Aramides albiventris*.

### Variation and taxonomy in the South American component

Two taxa can be identified in the South American component of the *Aramides cajaneus* species complex: *Aramides cajaneus* (Statius Müller, 1776), *sensu stricto*, found from Costa Rica south to Uruguay and northern Argentina; and *Aramides avicenniae* Stotz, 1992, found in a small part of the coast of southeastern Brazil.

**Figure 11.**
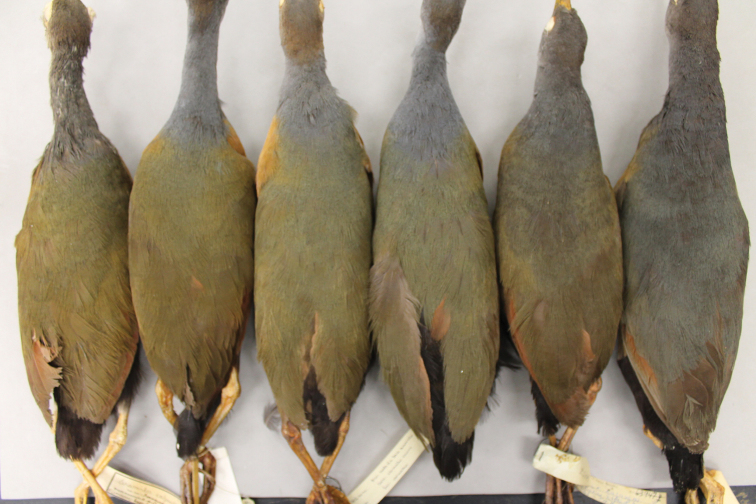
Specimens of *Aramides avicenniae* (the rightmost specimen) and *Aramides cajaneus* (all others) from Brazil. Note the homogeneous grey coloration in the hindneck and back of *Aramides avicenniae*, while in *Aramides cajaneus* the back is always greener than the hindneck.

*Aramides avicenniae* is distinguished from *Aramides cajaneus* by its gray, instead of green, back and its more greenish-gray upper wing-coverts. Throughout the distribution of *Aramides cajaneus*, back color is somewhat variable and even tends towards grayish-green in several specimens from the southwestern part of its distribution and from the northern coast of São Paulo state, not far from the range of *Aramides avicenniae*. Nevertheless, when specimens of *Aramides avicenniae* and even the grayest-backed specimens of *Aramides cajaneus* are placed side-by-side, there is a clear discontinuity in the color of their backs (Figure 11). In specimens of *Aramides cajaneus* from Ilha dos Búzios, Ilha Alcatrazes and Ubatuba, on the northern coast of São Paulo, the hindneck and upper back are clearly of different colors, even if in some of them the back is darker than the average in *Aramides cajaneus*. On the other hand, the upper back and the neck are display the same tone of gray in specimens from the São Paulo coast south of Santos (*Aramides avicenniae*). These patterns demonstrate that *Aramides avicenniae* is not merely the end of a cline, nor a variation of *Aramides cajaneus*, and it is hereby regarded as a full species.

Both sexes of *Aramides cajaneus* (*sensu stricto*) have significantly smaller bill height than *Aramides avicenniae*, and males have significantly smaller bill width. In addition, the two are significantly smaller than *Aramides albiventris* in tail length, tarsus length and bill length of both sexes. (Tables 1–3).

**Table 2.** Results of the ANOVA (parametric) or Kruskall-Wallis (KW; non-parametric) tests comparing the three recognized species. Significant p-values (< 0.05) are in italics.

	Wing		Tail		Tarsus		Bill heigth		Bill width		Bill length	
	Males	Females	Males	Females	Males	Females	Males	Females	Males	Females	Males	Females
Test	KW	KW	ANOVA	KW	ANOVA	KW	KW	KW	ANOVA	KW	KW	KW
p	0.1108	0.6421	*<0.0001*	*0.0019*	*<0.0001*	*<0.0001*	*0.0002*	*0.0015*	*0.0036*	0.1302	*<0.0001*	*<0.0001*

**Table 3.** Results of the post-hoc pairwise comparison tests (Tukey or Dunns) between the recognized taxa. Ns: not significant (p>0.05); *: 0.05>p>0.01; **: 0.01>p>0.001; ***: p<0.001.

	Wing		Tail		Tarsus		Bill heigth		Bill width		Bill length	
	Males	Females	Males	Females	Males	Females	Males	Females	Males	Females	Males	Females
*cajaneus* × *avicenniae*	ns	Ns	ns	ns	ns	ns	***	**	**	ns	ns	ns
*cajaneus* × *albiventris*	ns	Ns	***	**	***	***	ns	ns	ns	ns	***	***
*avicenniae* × *albiventris*	ns	Ns	**	**	***	**	ns	ns	ns	ns	*	**

Bangs and Penard (1918) described *Aramides cajanea latens*, from the islands of San Miguel and Viveros, in the Pearl Island archipelago off the Pacific coast of Panama. It was distinguished from *Aramides cajaneus* by its smaller size and overall paler plumage. *Aramides cajaneus morrissoni* was described from the islands of San José and Pedro González, in the same archipelago, by Wetmore (1946), as being similar to *latens*, but told apart by its darker back and hindneck. However, all the specimens from the Pearl Islands examined, including the types of both subspecies (MCZ 114297 and USNM 376059, respectively), fall within the variation observed for *Aramides cajaneus* and these names are thus treated as junior synonyms. These synonyms of *Aramides cajaneus*, along with *Rallus chiricote*, *Aramides cajanea venezuelensis*, *Aramides cajanea peruviana*, *Aramides cajanea salmoni* and *Aramides cajanea grahami*, are probably the result of overemphasis on minor individual plumage variations and lack of adequate and geographically comprehensive sampling.

### The distribution of *Aramides cajaneus* in southeastern Brazil

**Figure 12.**
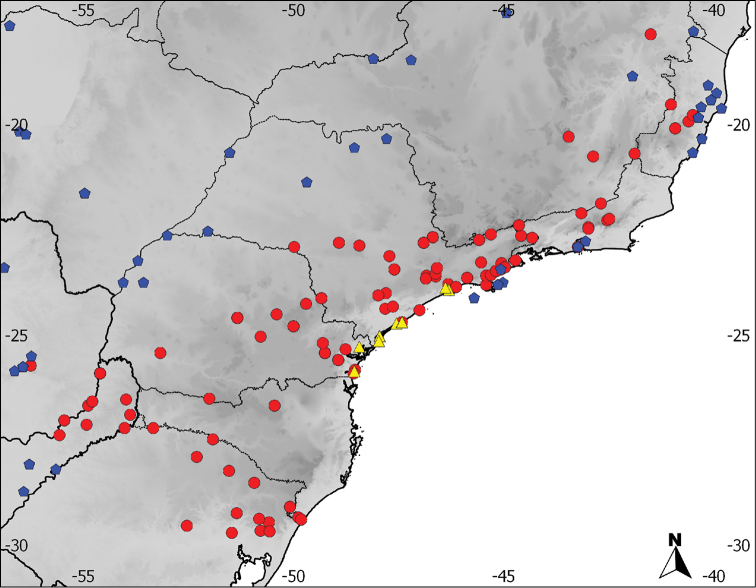
Distribution of *Aramides cajaneus* (blue), *Aramides avicenniae* (yellow) and *Aramides saracura* (red) in southeastern Brazil. Note the parapatric distribution pattern.

When the distributions of *Aramides avicenniae*, *Aramides cajaneus* and their congener *Aramides saracura* (Spix, 1825) are mapped together, it is notable they have almost perfectly parapatric distributions, a pattern never before remarked on. Contrary to what is indicated in several reference works (e. g. Ripley 1977, Taylor 1996, Taylor 1998, Erize et al. 2006, Sigrist 2009), *Aramides cajaneus* is absent from an extensive part of interior southeastern Brazil and from the Argentine province of Misiones. This area corresponds almost exactly to the distribution of *Aramides saracura* (Figure 12). *Aramides saracura* and *Aramides cajaneus* (or its substitute *Aramides avicenniae*) both occur on the coast of this region, but in that case *Aramides cajaneus* and *Aramides avicenniae* are mainly found in mangroves, a habitat not occupied by *Aramides saracura* (Taylor 1998). Even though *Aramides saracura* is usually considered more of a forest dweller than *Aramides cajaneus* (Taylor 1998), it is possible that their ecological preferences are not different to the point of allowing sympatry. A hypothesis derived from this distribution pattern is that *Aramides saracura* might have been the implied in the differentiation between *Aramides avicenniae* and *Aramides cajaneus*. Its presence might have acted as an ecological barrier between inland and coastal populations of *Aramides cajaneus*, leading to a process of peripatric speciation that culminated with the emergence of *Aramides avicenniae*.

### A clarification regarding the date of description of *Aramides plumbeicollis*

Even though we do not recognize *Aramides plumbeicollis* as a valid taxon, a clarification is needed regarding this name, given that it is nomenclaturally available and most references have a wrong publication date for it. Hellmayr and Conover (1942) cite the description of *Aramides plumbeicollis* as “*Anal. Mus. Nac. Costa Rica, 2, p. 3, 1888*”, and a similar citation is given by Ripley (1977). Taylor (1996), Taylor (1998) and Dickinson and Remsen (2013), also have the year as 1888, but without the full reference. However, a careful examination of the relevant publications reveals that the name *Aramides plumbeicollis* was first published, under the rules of the Code, only in 1892.

The name *Aramides plumbeicollis* was first used in a catalogue of the birds of Costa Rica in tome 1 of the Anales del Museo Nacional—República de Costa Rica (Zeledón 1888: 131). In this publication the name is not associated with any definition or description of the taxon to which it refers. There is only a footnote that reads: “This species is described on page 3, Tome II of these Annals, Year 1888” (our translation from the Spanish original). According to Article 12 of the Code, names published before 1931 without a description or definition are considered available as long as they are associated with an indication of the animal they refer to. A reference to a past publication fulfills this requirement, but a reference to a future publication does not. Therefore, *Aramides plumbeicollis* Zeledón, 1888, as it appears in this catalogue, is a *nomen nudum*.

Subsequent authors always gave 1888 as the date of the species’ description, probably assuming, based on Zeledón’s (1888) footnote, that *Aramides plumbeicollis* was indeed described in tome 2 of the Anales del Museo Nacional—República de Costa Rica. However, no exemplars of this publication could be found in the library of the Museo Nacional de Costa Rica (Adelina Jara, librarian at the Museo Nacional de Costa Rica, pers. comm.) and this volume was actually never published (Anonymous 1892, Chaves and Bolaños 2011). In fact, following tome 1, the publication was merged with the Anales del Instituto Fisico-Geografico Nacional de Costa Rica, giving rise to a new series titled Anales del Instituto Fisico-Geografico y del Museo Nacional de Costa Rica. However, the numbering of this new series continued with that of the Anales del Instituto Fisico-Geografico Nacional, and its first tome, published in 1892, but referring to 1890, is tome 3 (Anonymous 1892). It is on page 134 of this tome that the description of *Aramides plumbeicollis* is found, and this is the first time in which that name is made available under the rules of the Code.

## Taxonomic accounts

### Aramides cajaneus

Taxon classificationAnimaliaGruiformesRallidae

(Statius Müller, 1776)

Fulica Cajanea Statius Müller, 1776. Natursystems Supplements, p. 119. Based on “La Grande Poule d’Eau de Cayenne” from Buffon (1781), which is illustrated in the Planches Enlumineés d’Histoire Naturelle by L. J. M. Daubenton (plate 352). Type locality: “Caienne” (Cayenne, French Guyana).
Fulica major Boddaert, 1783. Table des Planches Enlumineéz d’Histoire Naturelle de M. D’Aubenton, p. 21. Based on Buffon’s (1781) “La Grande Poule d’Eau de Cayenne”.
Fulica cayennensis Gmelin, 1789. Systema Naturae, 13th edition, v. 1, part 2, p. 700. Based on Latham’s (1785) “Cayenne Gallinule” and Buffon’s (1781) “La Grande Poule d’Eau de Cayenne”. Type locality: “Guianae et Cayennae”.
Fulica ruficollis Gmelin, 1789. Systema Naturae, 13th edition, v. 1, part 2, p. 700. Based on Latham’s (1785) “Black-Bellied Gallinule”. Type locality: “Cayenna”.
Rallus chiricote Vieillot, 1819. Nouveau Dictionnaire d’Histoire Naturelle, v. 28, p. 551. Based on Azara’s (1805) “Chiricóte”. Type locality: “Paraguay”. Azara’s “Chiricóte aplomado” which Vieillot (1819) considered a variant of his *Rallus chiricote*, is actually *Aramides saracura*.
Rallus maximus Vieillot, 1819. Nouveau Dictionnaire d’Histoire Naturelle, v. 28, p. 555. Based on Latham’s (1785) “Cayenne Gallinule”, Gmelin’s (1789) *Fulica cayennensis* and Buffon’s (1781) La Grande Poule d’Eau de Cayenne”. Type locality: “Cayenne et [...] Guyane”.
Gallinula ruficeps Spix, 1825. Avium Species Novae, tome 2, p. 74 and plate 96. Type specimen in the Munich museum, not examined. Type locality: “Provincia Rio de Janeiro” (Rio de Janeiro state, Brazil).
Rallus hydrogallina Lesson, 1831. Traité d’Ornithologie, p. 536. Based on Gmelin’s (1789) *Fulica cayennensis* and Buffons’s (1781) “La Grande Poule d’Eau de Cayenne”. Type locality: “Cayenne” and “Brésil”. The supposed juvenile with slate underparts (“dessous du corps ardoisé”) is not *Aramides cajaneus*.
Aramides gutturalis Sharpe, 1894. Catalogue of the Birds in the British Museum, v. 23, p. 57 and plate 5. Holotype, examined: BMNH 1843.5.24.134, “South America”. The specimen’s oldest label bears the word “Lima”. However, no species of *Aramides* is known to occur in the vicinity of Lima, Peru. If this is indeed the locality meant, then it is likely that it represents simply the port from where the skin was shipped to Europe, rather than the actual place where it was collected.
Aramides cajanea venezuelensis Cory, 1915. Field Museum of Natural History Ornithological Series, v. 1, n. 8, p. 296. Holotype, examined: FMNH 34472, adult male, “Encontrados, Venezuela” (Zulia state).
Aramides cajanea peruviana Cory, 1915. Field Museum of Natural History Ornithological Series, vol. 1, n. 8, p. 296. Holotype, examined: FMNH 44019, adult female, “Moyabamba, Peru” (San Martín department).
Aramides cajanea latens Bangs & Penard, 1918. Bulletin of the Museum of Comparative Zoology, v. 62, p. 41. Holotype, examined: MCZ 114297, adult female, “San Miguel Island, Bay of Panama” (known now as Isla del Rey, in the Las Perlas archipelago).
Aramides cajanea salmoni Chubb, 1918. Bulletin of the British Ornithologists’ Club, v. 38, p. 48. Holotype, examined: BMNH 89.11.20.50, “Remedios, Antioquia, Colombia”.
Aramides cajanea grahami Chubb, 1919. The Ibis, 11th series, v. 1, n. 1, p. 53. Holotype, examined: BMNH 45.8.25.56, “Pará, Brazil”.
Aramides cajanea morrisoni Wetmore, 1946. Proceedings of the Biological Society of Washington, v. 59, p. 50. Holotype, examined: USNM 376059, adult male, “San José Island, Archipiélago de las Perlas” (Panama).

#### Diagnosis.

Nuchal spot very dark grayish-brown 10YR 3/2, sometimes duller or, very rarely, absent. Back entirely green. No white or pale feathers whatsoever on the lower chest. Basic phrase of the song bisyllabic (see details above).

#### Distribution.

Pacific side of Costa Rica; Panama (including the Pearl Islands); Colombia (except the Chocó region, west of the Andes); Venezuela; the Guianas; Ecuador, Peru and Bolivia east of the Andes; Brazil (except a section of the coast where it is replaced by *Aramides avicenniae*, and some inland parts of the states of São Paulo, Paraná, Santa Catarina and Rio Grande do Sul, where it is replaced by *Aramides saracura*; see above); southeastern Paraguay; Uruguay; and extreme northwestern and northeastern Argentina (Jujuy, Salta, Corrientes, Entre Rios and Buenos Aires provinces) (Figures 1 and 2).

### Aramides avicenniae

Taxon classificationAnimaliaGruiformesRallidae

Stotz, 1992

Aramides cajanea avicenniae Stotz, 1992. Bulletin of the British Ornithologists’ Club, v. 112, n. 4, p. 232. Holotype, examined: MZUSP 67212, adult male, “Iguape, São Paulo, Brazil”.

#### Diagnosis.

Brown nuchal spot absent or very inconspicuous. Gray upper-back (mantle) and hindneck, with greenish-gray upper wing-coverts. No white or pale feathers whatsoever on the lower chest. Basic phrase of the song bisyllabic (see details above).

#### Distribution.

Coastal Brazil from Santos, São Paulo state, south to Guaratuba Bay, Paraná state (Figures 1 and 12). A single USNM specimen from Santa Catarina state is also mentioned by Bangs (1907). According to him, it agrees completely with BMNH 89.11.20 from the Paraná coast, which we examined and is a typical *avicenniae*. The USNM specimen mentioned by Bangs could not be examined by us, but indicates that the species’ distribution may extend further south to at least Santa Catarina.

### Aramides albiventris

Taxon classificationAnimaliaGruiformesRallidae

Lawrence, 1868

Aramides albiventris Lawrence, 1868. Proceedings of the Academy of Natural Sciences of Philadelphia, v. 19, p. 234. Syntypes, examined: AMNH 45656, “British Honduras” (=Belize) and AMNH 45657, “Guatemala”.
Aramides plumbeicollis Zeledón, 1892. Anales del Instituto Físico Geográfico y del Museu Nacional de Costa Rica, tome 3, p. 134. Holotype, examined: USNM 113603, adult male, “Jiménez, lugar situado sobre la línea del ferrocarril en la planicie del Atlántico como á 56 millas del puerto de Limón, y á una altura como de 700 pies sobre el nível del mar”, Costa Rica.
Aramides albiventris mexicanus Bangs, 1907. The American Naturalist, v. 41, n. 483, p. 185. Holotype, examined: MCZ 110281, “Buena Vista, Vera Cruz, Mexico”.
Aramides plumbeicollis pacificus Miller & Griscom, 1921. American Museum Novitates, n. 25, p. 11. Holotype, examined: AMNH 143684, adult male, “Tipitapa, Nicaragua”.
Aramides vanrossemi Dickey, 1929. The Condor, v. 31, p. 33. Holotype, examined: UCLA 18750, adult male, “Barra de Santiago, Ahuachapan, El Salvador”.

#### Diagnosis.

Strong brown nuchal spot (Very Dark Brown 7.5YR 2.5/3). Basic phrase of the song containing at least nine notes (see above for details).

#### Distribution.

From the Caribbean side of Costa Rica northwards throughout Central America to southwestern Tamaulipas state, in Mexico (Figures 1 and 2).

### Notes on plumage variation in other species of Aramides

***Aramides ypecaha***

This species has a seemingly disjunct distribution, being found in central Brazil, especially along the Araguaia and São Francisco river valleys, as well as, further south, in southern Brazil, Paraguay, Uruguay and northeastern Argentina, but with no records from the extensive intermediate area. Nevertheless, no morphological differentiation has been described between these two populations. Based on 66 specimens, the only difference observed was that specimens from the northern population have slightly grayer and darker backs than those from the southern population (5Y 4/3 versus 2.5Y 4/3, respectively). There is, however, considerable variation within each population, and the differences are too subtle to allow a safe, consistent diagnosis. In addition, the species’ peculiar distribution needs to be further investigated before further taxonomic or evolutionary inferences can be made.

***Aramides wolfi***

**Figure 13.**
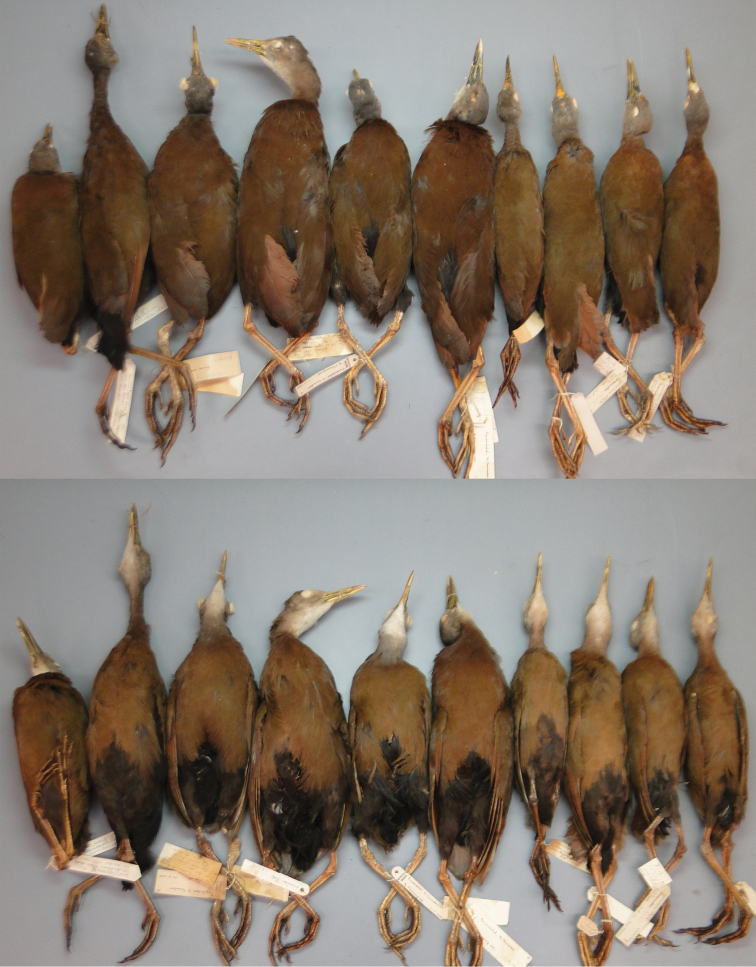
A series of *Aramides wolfi* in the AMNH. The six leftmost specimens, with a stronger coloration, are from northwestern Ecuador, while the four specimens to the right, from southwestern Ecuador, have a paler plumage.

This species is considered Vulnerable in the IUCN Red List (BirdLife International 2012). It is also the *Aramides* with the most restricted distribution; only found west of the Andes from southwestern Ecuador north to the Chocó department of Colombia. From the 26 skins analyzed, we found that specimens from central and southern Ecuador are much paler than those from Colombia and the departments of Pichincha and Esmeraldas, in northern Ecuador (Figure 13). Southern specimens have pale greenish-brown backs (7.5YR 3/2 to 7.5YR 3/4), while northern ones are strong reddish-brown (5YR 2.5/2) (Figure 13). The underparts of northern specimens are also darker and redder but this is subtler than the difference in the upperparts. Where the two variants approach each other, in the region of Pichincha, Manabi and Esmeraldas, intermediates are present.

This variation coincides with a notable climatic gradient; from one of the most humid regions on Earth, in southwestern Colombia, to semi-arid conditions in southwestern Ecuador. This is consistent with Gloger’s rule, according to which animal populations from humid regions tend to be darker and more pigmented than those from dry climates (Gloger 1833, Zink and Remsen 1986). The mechanisms behind Gloger’s rule are not necessarily genetic (Zink and Remsen 1986, and see Beebe 1907, Slagsvold and Lifjeld 1985), and thus we refrain from making any taxonomic or evolutionary inferences based on the variation observed in *Aramides wolfi*, and suggest that further investigations are required to determine the mechanisms responsible for it and the taxonomic implications thereof.

***Aramides mangle***

This species occurs along the coast of Brazil from Pará to Paraná, with some inland records in northeastern Brazil which indicate occurrence of migratory movements (Redies 2010, Marcondes et al. 2014). Two plumage variants were observed in it. The coloration pattern is the same in the two, but in one variant the whole plumage is much paler. Even though pale specimens come mainly from northeastern Brazil, there is no geographical segregation between the variants, as dark-plumaged birds also occur in that region (e. g. FMNH 403199, from Piauí, and MPEG 67808, from Maranhão). Indeed both forms have even been collected in the same locality (MNHN 1971.786 and 1971.787, from Exu, Pernambuco). Given this lack of geographical pattern, the plumage variation in *Aramides mangle* is considered intraspecific and taxonomically uninformative. Its exact nature remains uncertain, but we hypothesize either that (1) the pale individuals are juveniles, although there are no notes on any of the specimen labels regarding their age. (2) Dark and paler specimens represent an intraspecific polymorphism with two distinct, discrete plumage morphs or phases. Or (3) that paleness is due to feather wear, possibly related to abrasion or exposure to sunlight.

## Supplementary Material

XML Treatment for Aramides cajaneus

XML Treatment for Aramides avicenniae

XML Treatment for Aramides albiventris

## Supplementary materials

Supplementary material 1 Specimens examined Data type: measurement Explanation note: A list of specimens (skins) examined of this study. This dataset is made available under the Open Database License (http://opendatacommons.org/licenses/odbl/1.0/). The Open Database License (ODbL) is a license agreement intended to allow users to freely share, modify, and use this Dataset while maintaining this same freedom for others, provided that the original source and author(s) are credited.Rafael Sobral Marcondes

Supplementary material 2 Recordings examined Data type: tape recording Explanation note: A list of tape recordings examined for this study. This dataset is made available under the Open Database License (http://opendatacommons.org/licenses/odbl/1.0/). The Open Database License (ODbL) is a license agreement intended to allow users to freely share, modify, and use this Dataset while maintaining this same freedom for others, provided that the original source and author(s) are credited.Rafael Sobral Marcondes
